# Association between Secondhand Smoke and Allergic Diseases in Korean Adolescents: Cross-Sectional Analysis of the 2019 KYRBS

**DOI:** 10.3390/healthcare11060851

**Published:** 2023-03-14

**Authors:** Mi-Jin Choi, Jong Park, So-Yeong Kim

**Affiliations:** 1 Department of Health Administration, College of Health and Welfare, Dongshin University, 67, Dongsindae-gil, Naju-si 58245, Republic of Korea; 2 Department of Preventive Medicine, College of Medicine, Chosun University, 309, Pilmun-daero, Dong-gu, Gwangju 61452, Republic of Korea

**Keywords:** adolescents, allergic disease, exposure, place, secondhand smoking

## Abstract

This study aimed to determine whether secondhand smoking (SHS) in adolescents is associated with allergic diseases. Data from the 2019 Korea Youth Risk Behavior Survey were used (N = 37,848 Korean adolescents: 19,114 boys and 18,734 girls). This study assessed SHS exposure using questionnaire data, which included information, such as exposure to SHS and allergic diseases. Multiple logistic regression analysis was used to estimate the association between exposure to SHS and allergic diseases. The results showed that exposure to SHS in schools and public places, exposure for 1–3 days (OR = 1.158, 95% CI, 1.077–1.246), 4–6 days (OR = 1.308, 95% CI, 1.190–1.438), and everyday (OR = 1.306, 95% CI, 1.187–1.437), and exposure in one, two, or three places were significantly associated with allergic diseases (one OR = 1.226, 95% CI, 1.169–1.128, two OR = 1.289, 95% CI, 1.222–1.360 and three OR = 1.282, 95% CI, 1.177–1.395). The present results show that exposure to SHS occurs in various places and at various frequencies, and it is associated with allergic diseases in Korean adolescents.

## 1. Introduction

Secondhand smoking (SHS) refers to the smoke from the tip of a cigarette or smoke exhaled by a smoker. Recently, we have discussed the inclusion of thirdhand smoke rather than direct exposure to cigarette smoke [1,2]. SHS is one of the causes of indoor air pollution, which is known to cause cardiovascular diseases, diabetes, lung cancer, and allergic diseases, with an estimated global mortality of 1% [3,4]. In recent years, various anti-smoking policies have been introduced to protect non-smokers, and Korea has implemented various policies to reduce SHS in accordance with the World Health Organization Framework Convention on Tobacco Control [5]. In Korea, the National Health Promotion Act was enacted in 1995 to designate non-smoking areas in facilities, such as medical institutions and large buildings. Smoking in all confectioneries, rest areas, general restaurants, and other places was banned in Korea on 1 January 2015. Based on the revised anti-smoking law, indoor sports facilities were also designated as non-smoking facilities [6]. Consequently, exposure to SHS has been decreasing with the extension of non-smoking areas and various public relations and campaigns.

Despite the widespread designation of non-smoking areas, adolescents are continually being exposed to SHS in various places other than their homes, such as schools, roads, and public places.

Adolescence is a period where various changes, such as the development of secondary sexual characteristics, physical growth, and cognitive and emotional changes, occur rapidly, and self-consciousness is not fully matured [7,8]. Daily, adolescents are involuntarily exposed to SHS, which causes greater damage than that caused in adults [2]. Continual exposure to SHS in adolescents can negatively affect neurocognitive abilities, increase their likelihood of becoming smokers, and lead to nicotine addiction [9,10]. Allergic diseases are associated with various factors, such as genetic and immunological factors, lifestyle, and age, but the main cause of these diseases is not known [11]. Several recent studies have shown that harmful substances in tobacco smoke are associated with allergic diseases [12].

SHS increases the prevalence of asthma, an allergic disease [13], and studies by He et al. have shown a positive association between prenatal and postpartum SHS exposure and the incidence of childhood asthma, asthma-like syndromes, and wheezing [12,14]. However, the participants of these studies were mainly adults or children, and there have been insufficient studies recruiting adolescents. There were studies on exposure to SHS at home, but studies on exposure in schools and public places where adolescents mainly live were insufficient. The purpose of this study was to investigate the relationship between SHS exposure and allergic diseases among Korean adolescents. In this study, data from the 15th Korean Adolescent Risk Behavior Survey were analyzed to evaluate the relationship between exposure to SHS in homes, schools, and public institutions in Korea and allergic diseases.

## 2. Materials and Methods

### 2.1. Data Source and Study Participants

The study used data from the 15th Korea Youth Risk Behavior Web-based Survey (KYRBS) in 2019, conducted by the Ministry of Education, the Ministry of Health and Welfare, and the Korea Centers for Disease Control and Prevention Agency (KDCA), The KYRBS is an annual cross-sectional survey to collect health information from Korean adolescents. To select all of Korea, the KYRBS selected 800 sample schools (400 middle and 400 high schools) using stratified cluster sampling methods. A sample class was randomly selected from each grade in the sample school, and a survey was conducted on all youths in the sample class [12]. This survey is an anonymous self-report online survey; 60,100 people were selected for the 2019 survey, and 57,303 people participated in the survey (response rate 95.3%). KYRBS protocol was approved by the institutional review board of the KDCA(2014-06EXP-02-P-A) and informed consent was obtained from all participants. Additional information is provided elsewhere [14].

### 2.2. Secondhand Smoking Status

The participants provided responses to the following questions: “How many days in the last 7 days did you inhale cigarette smoke in your home?”; “How many days in the last 7 days did you breathe in cigarette smoke that someone else smoked in indoor school areas (classrooms, toilets, hallways, etc.) designated as non-smoking areas?”; and “How many days in the last 7 days did you breathe in cigarette smoke from someone else in indoor non-smoking areas (stores, restaurants, shopping malls, concert halls, personal computer rooms, karaoke rooms, etc.), except for your home or school?”, which were classified into “none”, “1–3 days per week”, “4–6 days per week”, and every day.

### 2.3. Allergic Disease Status

Those who responded “yes” to the question, “Have you ever been diagnosed with ‘asthma’ by a doctor after you were born,” were classified as patients with asthma; those who responded “yes” to the question, “Have you ever been diagnosed with ‘allergic rhinitis’ by a doctor after birth?”, were classified as patients with allergic rhinitis; those who responded ‘yes’ to the question, “Have you ever been diagnosed with atopic dermatitis by a doctor after at any point in life?”, were classified as patients with atopic dermatitis; and those who responded with “I had at least one of these diseases” were classified as having allergic diseases.

### 2.4. General Characteristics

Non-smoker subjects were classified according to sex (male, female), residential area (large city, small and medium-sized city, county area) and were further reclassified according to the type of school (middle, high schools), academic record (high, middle, low), economic level (high, middle, low), and cohabitation type (family, others). Health status was classified according to the stress level, subjective health status (high, average, poor), obesity status (normal: BMI < 25, obesity: BMI ≥ 25), drinking status (yes, no), and history of sadness or desperation severe enough to interfere with their daily life (yes, no).

### 2.5. Health Behavior Characteristics

Health status was classified according to the stress level (high, middle, low), subjective health status (good, common, bad), obesity status (normal: BMI < 25, obesity: BMI ≥ 25), and drinking status (yes, no). Those who responded “yes” to the question, “In the last 12 months, have you ever felt so sad or hopeless that it stopped you from doing your daily activities for a full 2 weeks?” were classified as depressed.

### 2.6. Statistical Analysis

The data from the KYRBS were analyzed using a weighted complex sampling analysis to ensure that the samples included in the KYRBS can represent the population. The characteristics of the subjects were analyzed using frequency analysis. The allergic disease-related characteristics were analyzed using the Rao–Scott chi-square test modified from the Pearson chi-square test. A binary logistic regression analysis was used to investigate the association between SHS and allergic diseases. The significance level was set at *p* < 0.05. All analyses were performed using IBM SPSS 26.0 software (IBM Co., Armonk, NY, USA).

## 3. Results

### 3.1. Characteristics of the Subjects

Among all participants, 47.4% males and 52.6% females had allergic diseases, and the difference between the sexes was significant (*p* < 0.001). Regarding the school type, 48.4% of middle school students and 51.7% of high school students had allergic diseases, and the difference was significant (*p* < 0.001). Regarding their academic records, 54.1% of those with a high academic record, 48.9% of those with an average academic record, and 46.8% of those with a low academic record had allergic diseases, which showed a statistically significant difference (*p* < 0.001). Regarding the residential area, 50.4% of those in big cities, 50.4% of those in small and medium cities, and 45.7% of those in county areas had allergic diseases, which showed a statistically significant difference (*p* < 0.001). However, there was no difference in the prevalence of allergic diseases according to their economic level and cohabitation family type (Table 1).

### 3.2. Characteristics of Health Behavior

Among those with allergic diseases, 44.4% perceived considerable stress, while 53.4% perceived low levels of stress, and the difference was statistically significant (*p* < 0.001). Regarding subjective health status, 47.3% had good health, 54.2% had common health, and 61.4% had bad health, which indicated a significant difference. Of the participants, 49.9% had a normal weight and 51.3% were obese. Furthermore, 51.2% of the participants consumed alcohol, and 48.4% were non-drinkers (*p* < 0.001). Further, 53.6% reported feeling depressed in the last 12 months, while 48.5% reported that they did not feel depressed in the last 12 months, and the difference was statistically significant (*p* < 0.001). Of those with allergic diseases, 49.0% were exposed to SHS at home, 52.2% were exposed to SHS at school, and 50.9% were exposed to SHS in public places. Furthermore, 51.2% had not been not exposed to SHS in the last 1 week, 48.1% had been exposed to SHS for 1–3 days per week, 50.0% had been exposed to SHS for 4–6 days per week, and 50.2% had been exposed to SHS everyday (*p* < 0.001). Among the participants with allergic diseases, 47.2% had not been exposed to SHS in any of the three places, 49.8% had been exposed to SHS in one place, 54.1% had been exposed to SHS in two places, and 53.4% had been exposed to SHS in all the three locations, and the difference was statistically significant (*p* < 0.001) (Table 2).

### 3.3. Association of the Place and Frequency of SHS Exposure with Allergic Diseases in Adolescents

Logistic regression analysis was performed to investigate the effects of SHS on allergic diseases. The results showed that the odds ratio (OR) for the association between allergic diseases and SHS exposure was significantly increased for schools and public places (OR: 1.136 [95% CI = 1.081–1.193] and OR: 1.260 [95% CI = 1.209–1.313], respectively) compared to that for allergic diseases with no exposure to SHS smoke, which indicated a statistically significant association. In addition, the ORs for allergic diseases with SHS exposure for 1–3 days per week, 4–6 days per week, and everyday were higher (OR: 1.158 [95% CI = 1.077–1.246], OR: 1.308 (95% CI = 1.190–1.438], and OR: 1.306 [95% CI = 1.187–1.437], respectively) than that for allergic diseases with no SHS exposure for 1 week, which indicated a statistically significant association. Moreover, in terms of the frequency of SHS exposure (places), the ORs for allergic diseases with SHS exposure at one, two, and three places (OR: 1.226 [95% CI = 1.169–1.285], OR: 1.289 [95% CI = 1.222–1.360], and OR: 1.282 [95% CI = 1.177–1.395], respectively) were higher than that of allergic diseases with no SHS exposure, which indicated a statistically significant association (Table 3).

## 4. Discussion

This study aimed to investigate the association between SHS and allergic diseases using data from the KYRBS conducted in South Korea. The results revealed that frequent exposure to SHS and SHS exposure at various places was significantly associated with allergic diseases. Previous studies regarding the association between SHS and allergic diseases showed a high rate of smoking and a high prevalence of allergic diseases in adolescents exposed to SHS, which is consistent with the results of this study [15,16].

In this study, the OR for SHS exposure at home was 1.006 (95% CI = 0.996–1.047) compared with that for no SHS exposure at home, which was consistent with the results of a previous study investigating factors affecting SHS at home in different participants that reported a decrease in SHS at home [17]. This might be owing to the continual implementation of smoke-free regulations and related education, leading to an increase in public awareness that SHS can adversely affect family members; in this study, the OR for exposure to SHS in school was higher (OR 1.136 [95% CI = 1.081–1.193]) for allergic disease, compared to that for no exposure to SHS in school, and the OR for exposure to SHS in public places was higher than that for no exposure to SHS in school (OR 1.260 [95% CI = 1.209–1.313]). The participants with allergic disease who were exposed more to SHS in schools and public places were more prevalent than those with allergic diseases who were not exposed to SHS in public. These results are consistent with those of previous studies that have suggested that exposure to SHS is associated with allergic diseases [18,19]. According to the KYRBS, the rate of exposure to SHS in schools increased from 20.0% in 2018 to 21.6% in 2019, and that in public places increased from 51.4% in 2018 to 52.5% in 2019 [14]. Although schools and public places have been designated as non-smoking areas, the surveyed participants had been more exposed to SHS in schools and public places than in other places. A previous study reported that SHS levels also increased in non-smoking indoor areas adjacent to outdoor smoking areas, which may be caused by factors, such as wind speed and direction from the outdoor smoking areas [20].

Regarding exposure to SHS, the ORs for SHS exposure for 1–3 days, 4–6 days, and everyday in the last 7 days were higher for allergic diseases than that for no SHS exposure in the last 7 days. Regarding the frequency of SHS exposure (places), the ORs for SHS exposure in one, two, and three places were higher for allergic diseases than that for no exposure, indicating that more frequent SHS exposure was associated with allergic diseases. These findings are similar to those of previous studies that reported that more frequent SHS exposure was associated with a higher incidence of allergic diseases [21,22,23]. Adolescents spend most of their time in an academy, in a school, or with their peers rather than at home, leading to a longer time expenditure in outdoor activities. This results in high SHS exposure among adolescents [24,25]. Since reducing the frequency of SHS exposure can help in preventing allergic diseases and relieving symptoms of allergic diseases, educating active smokers is needed [26].

Adolescents have higher rates of metabolism and absorption of foreign substances than adults, although their development and growth are incomplete. Thus, their immune and respiratory systems may be vulnerable to exposure to harmful substances, such as cigarette smoke [3]. Therefore, it is important that adolescents are not exposed to SHS. To enhance public awareness about the harmful effects of SHS and reduce the associated damage, the Korean government created a slogan, “Say No, Save Life”, in 2008, and subsequently, efforts to reduce SHS have been made through various policies, public relations, and educational programs [27]. However, an increased SHS exposure in public places, as observed in this study, indicates that such efforts are limited in reducing SHS.

According to the studies of Tsai et al., SHS among U.S. nonsmokers has decreased considerably during the past two and a half decades, progress has stalled in recent years, and approximately one in four non-smokers remains exposed to this preventable health hazard [28]. Although comprehensive smoke-free laws are in place, continuous measures, the adoption of strong smoke-free rules, and educational interventions that warn of the dangers of SHS can further reduce SHS exposure, especially among vulnerable populations.

The study has some limitations. Since this was a cross-sectional study, the temporal and sequential association of each factor with allergic diseases was not established, and since the study analyzed secondary data from a self-administered survey, it has a limitation in the selection of variables. Moreover, since SHS can vary depending on environmental conditions, such as place, temperature, ventilation, and humidity, it is possible that the effects of SHS could have been underestimated. The conclusions and findings of this study cannot be generalized to different populations, and the study has all limitations and risks of bias inherent to surveys.

## 5. Conclusions

Despite the above limitations, this study is significant. Based on data from the KYRBS, a nationally representative data, this study confirmed that frequent SHS exposure in schools and public places is associated with allergic diseases in Korean adolescents. These findings indicate that while identifying the various conditions in which adolescents can be exposed to SHS, it is necessary to increase public awareness of SHS and implement institutional changes at the school, community, and country levels.

## Figures and Tables

**Table 1:** General characteristics.

Variable	Allergic Disease		Rao–Scott x²	*p*-Value
	No	Yes		
Total	19,114 (50.5)	18,734 (49.5)		
Sex				
Boys	9511 (52.6)	8397 (47.4)	74.332	<0.001
Girls	9603 (47.4)	10,337 (52.6)		
School				
Middle school	10,151 (51.6)	9257 (48.4)	28.363	<0.001
High school	8963 (48.3)	9477 (51.7)		
House ecomomic status				
High	7063 (49.1)	9257 (48.4)	4.323	0.013
Middle	9394 (50.7)	8936 (49.3)		
Low	2657 (49.2)	2707 (50.8)		
Preceived school record				
High	6638 (45.9)	7563 (54.1)	69.310	<0.001
Middle	5885 (51.1)	5518 (48.9)		
Low	6591 (53.2)	5653 (46.8)		
Type of residence				
Living with family	18,229 (50.0)	17,768 (50.0)	4.465	0.035
Other	885 (47.0)	996 (53.0)		
Residential area				
Big city	8285 (49.6)	8396 (50.4)	5.317	0.005
Small city	9204 (49.6)	9046 (50.4)		
Town	1625 (54.3)	1292 (45.7)		

Values are presented as numbers (weighted %). Rao–Scott chi-square test.

**Table 2:** Characteristics of health behavior.

Variable	Allergic Disease		Rao–Scott x²	*p*-Value
	No	Yes		
Total	19,114 (50.5)	18,734 (49.5)		
Stress status				
High	3487 (55.6)	2696 (44.4)	69.437	<0.001
Middle	7929 (51.0)	7.341 (49.0)		
Low	7698 (46.6)	8697 (53.4		
Subjective health status				
Good	13,704 (52.7)	11,961 (47.3)	158.998	<0.001
Common	4179 (45.8)	4829 (54.2)		
Bad	1231 (38.6)	1939 (61.4)		
Obesity				
Normal (BMI < 25)	15,687 (50.1)	15,283 (49.9)	2.684	0.102
Obesity (BMI ≥ 25)	2782 (48.7)	2808 (51.3)		
Drinking				
No	11,438 (51.6)	10,732 (48.4)	12.174	0.001
Yes	7676 (48.8)	8002 (51.2)		
Depression				
No	13,583 (51.5)	12,471 (48.5)	83.465	<0.001
Yes	5531 (46.4)	6263 (53.6)		
SHS place status				
At home				
No	9565 (48.8)	9838 (51.2)	18.319	<0.001
Yes	9549 (51.0)	8896 (49.0)		
At school				
No	13,290 (50.9)	12,525 (49.1)	27,517	<0.001
Yes	5824 (47.8)	6209 (52.2)		
At public place				
No	4402 (52.8)	3798 (47.2)	32.192	<0.001
Yes	14,712 (49.1)	14,936 (50.9)		
SHS exposure status/week				
No	9565 (48.8)	9838 (51.2)	8359	<0.001
1–3 day/week	5555 (51.9)	4978 (48.1)		
4–6 day/week	1713 (50.0)	1645 (50.0)		
Everyday	2281 (49.8)	2273 (50.2)		
SHS exposure frequency (place)				
No	4402 (52.8)	3798 (47.2)	23.229	<0.001
1 place	10,876 (50.2)	10,534 (49.8)		
2 place	2301 (45.9)	2642 (54.1)		
3 place	1537 (46.6)	1760 (53.4)		

BMI, body mass index; SHS, secondhand smoking. Values are presented as frequencies (weighted %). Rao–Scott chi-square test.

**Table 3:** Association of the secondhand smoking exposure place and frequency with allergic diseases in adolescents.

Variable	Odds Ratio (95% Confidence Interval) *
Place of SHS exposure	
At home	
No	1.000
Yes	1.006 (0.996–1.047)
At school	
No	1.000
Yes	1.136 (1.081–1.193)
At public place	
No	1.000
Yes	1.260 (1.209–1.313)
SHS exposure status/week	
No	1.000
1–3 day/week	1.158 (1.077–1.246)
4–6 day/week	1.308 (1.190–1.438)
Everyday	1.306 (1.187–1.437)
SHS exposure status/places	
No	1.000
1 place	1.226 (1.169–1.285)
2 places	1.289 (1.222–1.360)
3 places	1.282 (1.177–1.395)

OR, odds ratio; CI, confidence interval. By complex sampling analysis. * adjusted for sex, school, house economic status, perceived school record, residential area, stress status, subjective health status, drinking, and depression.

## Data Availability

Data are available on the official KRYBS website (https://www.kdca.go.kr/yhs/ accessed on 18 March 2020).
